# Associations of polygenic risk scores with risks of stroke and its subtypes in Chinese

**DOI:** 10.1136/svn-2023-002428

**Published:** 2023-08-28

**Authors:** Songchun Yang, Zhijia Sun, Dong Sun, Canqing Yu, Yu Guo, Dianjianyi Sun, Yuanjie Pang, Pei Pei, Ling Yang, Iona Y. Millwood, Robin G. Walters, Yiping Chen, Huaidong Du, Yan Lu, Sushila Burgess, Daniel Avery, Robert Clarke, Junshi Chen, Zhengming Chen, Liming Li, Jun Lv

**Affiliations:** 1 Department of Epidemiology & Biostatistics, School of Public Health, Peking University, Beijing 100191, China; 2 Department of Dermatology, Xiangya Hospital, Central South University, Changsha 410008, China; 3 Peking University Center for Public Health and Epidemic Preparedness & Response, Beijing 100191, China; 4 Key Laboratory of Epidemiology of Major Diseases (Peking University), Ministry of Education; 5 Fuwai Hospital Chinese Academy of Medical Sciences, Beijing 100730, China; 6 Medical Research Council Population Health Research Unit at the University of Oxford, Oxford OX3 7LF, United Kingdom; 7 Clinical Trial Service Unit & Epidemiological Studies Unit (CTSU), Nuffield Department of Population Health, University of Oxford, Oxford OX3 7LF, United Kingdom; 8 NCDs Prevention and Control Department, Suzhou CDC, Suzhou 215004, China; 9 China National Center for Food Safety Risk Assessment, Beijing 100738, China; 10 State Key Laboratory of Vascular Homeostasis and Remodeling, Peking University

**Keywords:** Stroke, Polygenic Risk Score, Chinese Population

## Abstract

**Background and purpose:**

Previous studies, mostly focusing on the European population, have reported polygenic risk scores (PRSs) might achieve risk stratification of stroke. We aimed to examine the association strengths of PRSs with risks of stroke and its subtypes in the Chinese population.

**Methods:**

Participants with genome-wide genotypic data in China Kadoorie Biobank were split into a potential training set (n=22,191) and a population-based testing set (n=72,150). Four previously developed PRSs were included, and new PRSs for stroke and its subtypes were developed. The PRSs showing the strongest association with risks of stroke or its subtypes in the training set were further evaluated in the testing set. Cox proportional hazards regression models were used to estimate the association strengths of different PRSs with risks of stroke and its subtypes (ischemic stroke [IS], intracerebral hemorrhage [ICH], and subarachnoid hemorrhage [SAH]).

**Results:**

In the testing set, during 872,919 person-years of follow-up, 8514 incident stroke events were documented. The PRSs of any stroke (AS) and IS were both positively associated with risks of AS, IS, and ICH (*P*<0.05). The HR for per standard deviation increment (HR_SD_) of PRS_AS_ was 1.10 (95% CI: 1.07-1.12), 1.10 (1.07-1.12), and 1.13 (1.07-1.20) for AS, IS, and ICH, respectively; The corresponding HR_SD_ of PRS_IS_ were 1.08 (1.06-1.11), 1.08 (1.06-1.11), and 1.09 (1.03-1.15). PRS_ICH_ was positively associated with the risk of ICH (HR_SD_ = 1.07, 95% CI: 1.01-1.14). PRS_SAH_ was not associated with risks of stroke and its subtypes. The addition of current PRSs offered little to no improvement in stroke risk prediction and risk stratification.

**Conclusions:**

In this Chinese population, the association strengths of current PRSs with risks of stroke and its subtypes were moderate, suggesting a limited value for improving risk prediction over traditional risk factors in the context of current GWAS underrepresenting the East Asian population.

## Introduction

Stroke is one of the leading causes of death and disease burdens globally.[1] Stroke includes two main subtypes, ischemic stroke (IS) and hemorrhagic stroke (HS). The latter could further be divided into intracerebral hemorrhage (ICH) and subarachnoid hemorrhage (SAH). With the accumulation of genomic data worldwide, the genetic background of stroke and its subtypes is gradually being revealed. Polygenic risk score (PRS), a method used to combine minor genetic effects across the whole genome, has been increasingly used in stroke research. Several studies based on European populations have developed PRSs for any stroke (AS) or IS and suggested their potential to improve risk prediction and risk stratification.[2–9] The incidence of stroke in China, especially ICH, is higher than in Western countries.[1] Recently, a PRS for AS was developed based on the Chinese population and showed similar association strength in predicting the risk of IS and HS.[10] However, IS and HS might have different etiological mechanisms.[11–13] Different stroke subtypes also have their specific genetic loci.[14] No study has specifically developed PRSs for subtypes of stroke in the Chinese population.

The present study was based on a sub-cohort with genomic data from the China Kadoorie Biobank (CKB). We aimed to examine the association strengths of PRSs with risks of stroke and its subtypes in the Chinese population.

## Methods

### Participants

CKB is an ongoing prospective study with 512,724 participants aged 30 to 79 enrolled from five urban and five rural regions in China between 2004 and 2008. Details of the study have been described elsewhere.[15] CKB had ethical approvals from the Ethical Review Committee of the Chinese Center for Disease Control and Prevention (Beijing, China) (approval notice: 005/2004) and the Oxford Tropical Research Ethics Committee, University of Oxford (UK) (reference: 025–04). All participants provided a written informed consent form.

**Figure 1:**
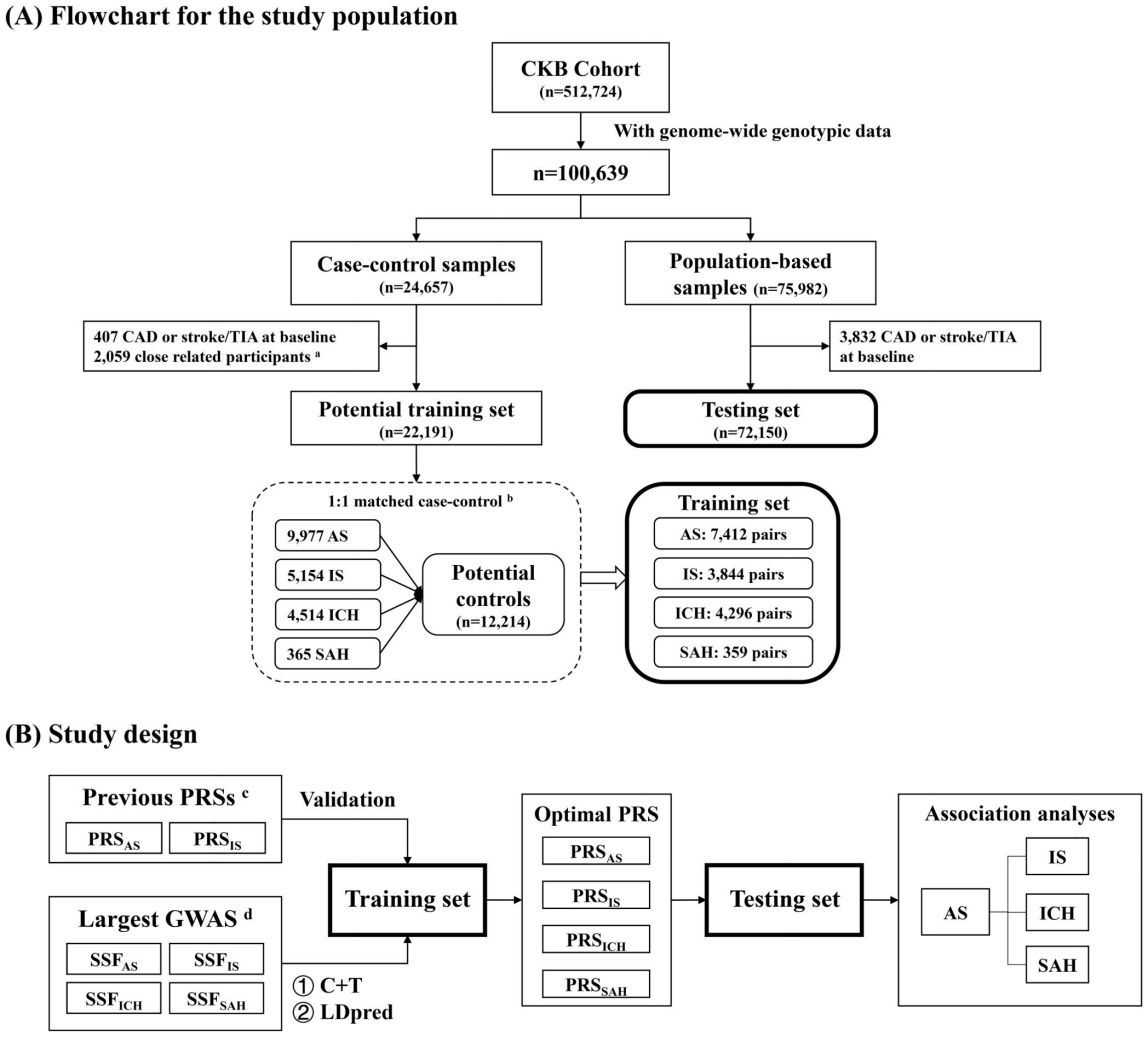
Overview of the present study (A) Flowchart for the study population; (B) Study design. The current study can be divided into four parts. (i) Validation of previous PRSs. (ii) Development of new PRSs. (iii) Identification of the optimal PRS for each outcome. (iv) Validation and evaluation of the optimal PRS for each outcome. Abbreviations: AS, any stroke; CAD, coronary heart disease; CKB, China Kadoorie Biobank; C+T, clumping & thresholding; GWAS, genome-wide association study; ICH, intracerebral hemorrhage; IS, ischemic stroke; PRS, polygenic risk score; SAH, subarachnoid hemorrhage; SSF, summary statistics file; TIA, transient ischemic attack. ^a^Participants that had a first or second-degree relative in the sample (kinship coefficient φ> 0.125) were removed by using PLINK 1.9. ^b^Please refer to supplemental methods for detailed procedures of case-control matching. ^c^See supplemental methods and supplemental table 3 for details. ^d^See supplemental methods and supplemental table 4 for details.

Among all CKB participants, there are 100,639 participants with genome-wide genotypic data. Of them, 24,657 participants were selected based on a case-control design nested within the cohort with the primary aim of studying CVD (“case-control samples”), which formed four matched-case-control training sets (figure 1A, supplemental methods, supplemental table 1, supplemental table 2). The other 75,982 participants were randomly selected from the entire CKB cohort (“population-based samples”); after excluding participants with self-reported coronary artery disease or stroke or transient ischemic attack at baseline (n=3,832), the remaining participants were used as a “testing set” (n=72,150) (figure 1A, supplemental methods).

### Study design

The current study can be divided into four parts (figure 1B). (1) Validation of previous PRSs. Four previously reported stroke-related PRSs were selected for validation.[2,4,5,10] (2) Development of new PRSs. Clumping & thresholding (“C+T”) and LDpred[16] were used to develop new PRSs for stroke and its subtypes based on two genome-wide association studies with large sample sizes.[14,17] (3) Identification of the optimal PRS for each outcome. The performances of different PRSs in predicting each outcome were compared in the corresponding training sets. (4) Validation and evaluation of the optimal PRS for each outcome. We prospectively examined the associations between optimal PRSs and risks of stroke and its subtypes. We evaluated the impact of PRSs on the risk prediction improvement by adding the optimal PRS to traditional risk prediction models in the testing set.

### Assessment of traditional stroke risk factors

The baseline questionnaire collected information on sociodemographic characteristics, lifestyle behaviors, dietary habits, and personal and family medical history.[15] Traditional stroke risk factors considered in the present study included sex, age, systolic and diastolic blood pressure (SBP and DBP), smoking, body mass index (BMI), waist circumference, hypertension, diabetes, and family history of stroke. Details on the collection and definition of these variables have been described in our previous work.[18,19]

### Genetic data

At baseline, a 10 mL random blood sample was collected from each participant. Genotyping and imputation in this study were centrally conducted, with detailsprovided in our previous study.[19,20] Briefly, two custom-designed single nucleotide polymorphism (SNP) arrays (Affymetrix Axiom® CKB array) were used for genotyping. Imputation was performed based on haplotypes derived from the 1000 Genomes Project Phase 3. There were 9.54 million genetic variants with high reliability (supplemental figure 1).

### Polygenic risk scores

We searched the PGS Catalog,[21] PubMed, and Embase. Four previous stroke PRSs were selected for validation analyses (supplemental methods, supplemental table 3).[2,4,5,10] Meanwhile, we ran *gwasfilter* to filter genome-wide association studies (GWAS) from the GWAS Catalog (https://www.ebi.ac.uk/gwas/).[22,23] Based on ethnicity, sample size, and accessibility of the summary statistics file (SSF), we finally included 1 AS SSF, 2 SAH SSFs, 2 ICH SSFs, and 2 IS SSFs from two large-scale GWAS (supplemental methods, supplemental table 4).[14,17] Similar to our latest research,[19] we developed new PRSs by using two methods: clumping and thresholding (“C+T”) and LDpred[16] (supplemental methods).

### Ascertainment of stroke outcomes

All participants were followed up for morbidity and mortality since their baseline enrollment. Incident events were identified by linking with local disease and death registries and the national health insurance database and supplemented by active follow-up.[15] In the testing set, only 653 (0.91%) were lost to follow-up before censoring on December 31, 2018. Trained staff blinded to baseline information codedall events using the International Classification of Diseases, Tenth Revision (ICD-10). Incident stroke events during the follow-up were defined as I60-I64, including SAH (I60), ICH (I61), other nontraumatic intracranial hemorrhage (I62), IS (I63), and unspecified stroke (I64). In the testing set, the events coded as I62 and I64 accounted for only 0.9% (n=76) and 3.5% (n=302) of all incident stroke events.

Since 2014, medical records of incident stroke cases have been retrieved and reviewed by qualified cardiovascular specialists blinded to baseline information. According to a previous study,[24] by October 2018, the reporting accuracy was 91.7%, 90.4%, and 82.7% for IS, ICH, and SAH;[24] the corresponding diagnostic accuracy was 93.1% (including silent lacunar infarction), 98.2%, and 98.1%, respectively.[24]

### Identification of the optimal PRS in the training set

In each training set, we used the conditional logistic regression model to measure the association of each PRS with the risk of the corresponding stroke outcome, stratified by the case-control pair, with the top 10 principal components of ancestry (PCA) and array versions as the covariates. We defined the optimal PRS as the PRS with the highest odds ratio (OR) per standard deviation (SD), as our previous study did.[19]

### Validation and evaluation of the optimal PRS in the testing set

In the testing set, we used the Cox regression model to measure the association of optimal PRSs with risks of stroke and stroke subtypes. The model was stratified by sex and ten study regions, with age as the time scale and adjusting for the top 10 PCA and array versions. We further adjusted for SBP, BMI, and family history of stroke in sensitivity analyses. We evaluated the proportional hazards assumptions by examining Schoenfeld residuals. Either non-existent or minimal deviations were observed. In subgroup analyses, the tests for multiplicative interaction were performed using likelihood ratio tests by comparing models with and without cross-product terms between the stratifying variable and PRS.

To evaluate the impact of PRS on risk prediction improvement, we defined the “CKB-CVD models” as the traditional risk prediction models, as our previous study did.[19] The “CKB-CVD models” distinguish risks of ischemic stroke and hemorrhagic stroke and have good discrimination without relying on blood lipids.[18] We added the PRS to traditional models to get a “PRS-enhanced model”. We assessed the discrimination performance by using Harrell’s C.[25] We used the net reclassification improvement (NRI) and integrated discrimination improvement (IDI) to evaluate model reclassification before and after the addition of PRS.[26]

The study adhered to the Polygenic Risk Score Reporting Standards (PRS-RS) and STROBE statement (Strengthening the reporting of observational studies in epidemiology) for cohort studies simultaneously (Supplemental file 2).[27,28] Analyses were done with Stata (V17.0, StataCorp) and R (V4.0.3). All statistical tests were two-sided with α = 0.05.

## Results

### Selection of the optimal PRSs in the training sets

In this study, four 1:1 matched training sets were defined to identify the optimal PRS for AS (7412 pairs), IS (3844 pairs), ICH (4296 pairs), and SAH (359 pairs) (figure 1, supplemental methods). Among the training sets, 72.7%, 61.6%, 77.9%, and 63.8% of the participants were from rural areas in China; 51.9%, 50.5%, 53.4%, and 38.4% of the participants were men, respectively. Among the cases, the median age of disease onset (25-75th percentile) was 65.3 (57.0-72.0), 64.1 (56.1-70.6), 65.9 (57.7-73.0), and 61.0 (53.8-69.2) years, respectively. Among all training sets, the proportion of the control group using the first version of the SNP array was lower than that of the case group (*P*<0.001) (supplemental table 2). The performance of PRS for AS and IS developed in previous studies was not better than that of the newly developed PRS in the present study (table 1, supplemental table 5). The optimal PRS for AS came from the LDpred method, and the optimal PRS for IS, ICH, and SAH came from the C+T method. The OR_SD_ (95% CI) of the optimal PRSs were 1.14 (1.10-1.18) for AS, 1.18 (1.13-1.24) for IS, 1.10 (1.05-1.15) for ICH, and 1.25 (1.06-1.47) for SAH (table 1, supplemental table 5).

### Associations of PRSs with stroke and its subtypes in the testing set

The testing set included 72,150 Chinese participants, of which 59.8% were women. The median age was 50.6 years in women and 51.9 years in men. During 872,919 person-years of follow-up (over 12 years on average), 8514 incident stroke events were documented, including 7507 IS, 1193 ICH, and 132 SAH (table 2). The correlations among the optimal PRSs were weak (all correlation coefficients < 0.2) (supplemental figure 2).

**Figure 2:**
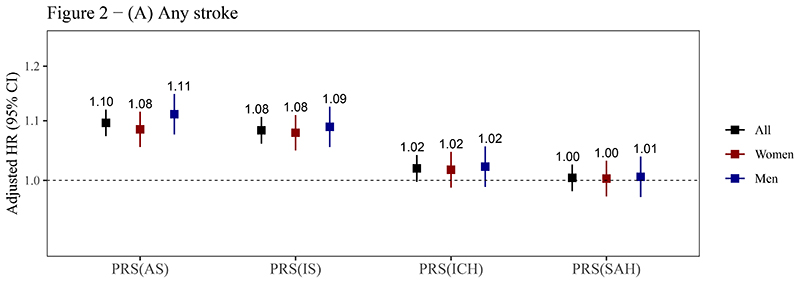

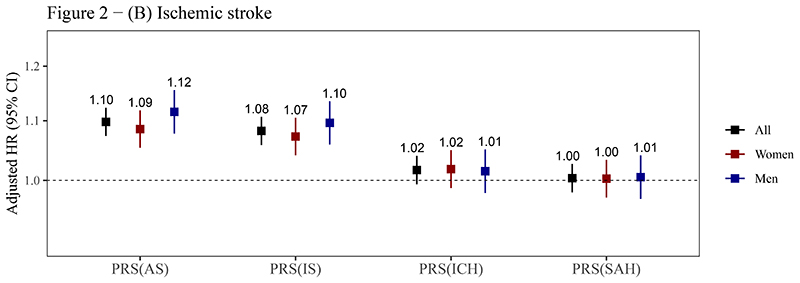

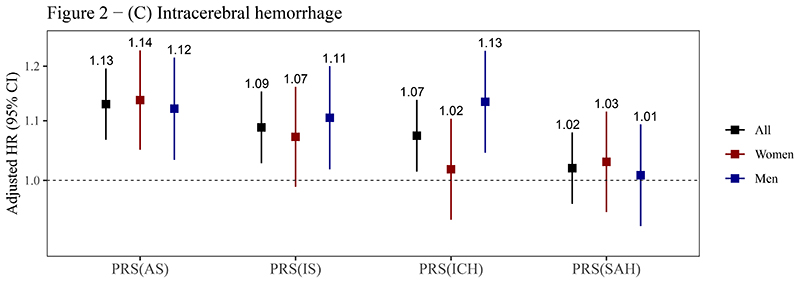

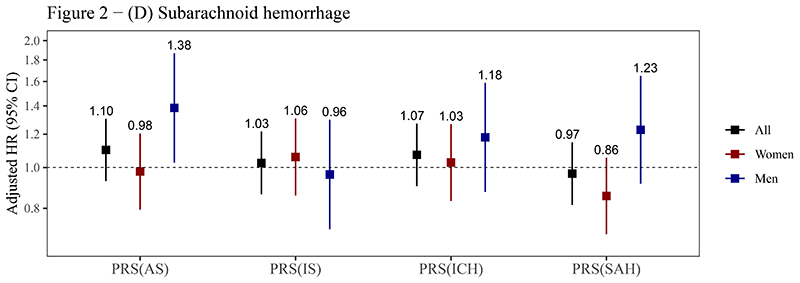
Associations of PRSs with risks of stroke and its subtypes (A) Any stroke; (B) Ischemic stroke; (C) Intracerebral hemorrhage; (D) Subarachnoid hemorrhage. Abbreviations: AS, any stroke; CI, confidence interval; HR, hazard ratio; ICH, intracerebral hemorrhage; IS, ischemic stroke; PRS, polygenic risk score; SAH, subarachnoid hemorrhage. The PRSs reported here are the optimal PRSs for stroke and its subtypes in the training sets (see table 1), which were standardized (zero mean, unit standard deviation) in the testing set. Cox models were stratified by sex and ten study regions and adjusted for the top 10 principal components of ancestry and array versions, with age as the time scale. The number above the closed square represents the HR. The number of stroke events in women and men has been reported in table 2. The vertical lines indicate 95% CIs.

The PRS_AS_ and PRS_IS_ were both positively associated with risks of AS, IS, and ICH (*P*<0.05). The HR_SD_ (95% CI) of PRS_AS_ were 1.10 (1.07-1.12), 1.10 (1.07-1.12), and 1.13 (1.07-1.20) for AS, IS, and ICH, respectively. The corresponding HR_SD_ (95% CI) of PRS_IS_ were 1.08 (1.06-1.11), 1.08 (1.06-1.11), and 1.09 (1.03-1.15) (figure 2, supplemental table 6). PRS_ICH_ was positively associated with the risk of ICH in the whole testing set (HR_SD_= 1.07), though it was not statistically significant in women (*P* for sex interaction = 0.056) (figure 2C). PRS_SAH_ was not associated with risks of any outcomes (figure 2). A strong association of PRS_AS_ with the risk of SAH (HR_SD_ = 1.38, 95% CI: 1.03-1.87) was observed in men but not in women (*P* for sex interaction = 0.055) (figure 2D).

In sensitivity analyses, the associations of PRSs with risks of stroke and its subtypes did not change significantly after additional adjustment for SBP, body mass index, and family history of stroke (supplemental table 6). In subgroup analyses, there was no strong evidence supporting a different association strength across subgroups for IS and ICH after considering multiple testing (*P* for interaction > 0.05/8) (supplemental figure 3, supplemental figure 4).

### Addition of the optimal PRS to traditional risk prediction models

**Figure 3:**
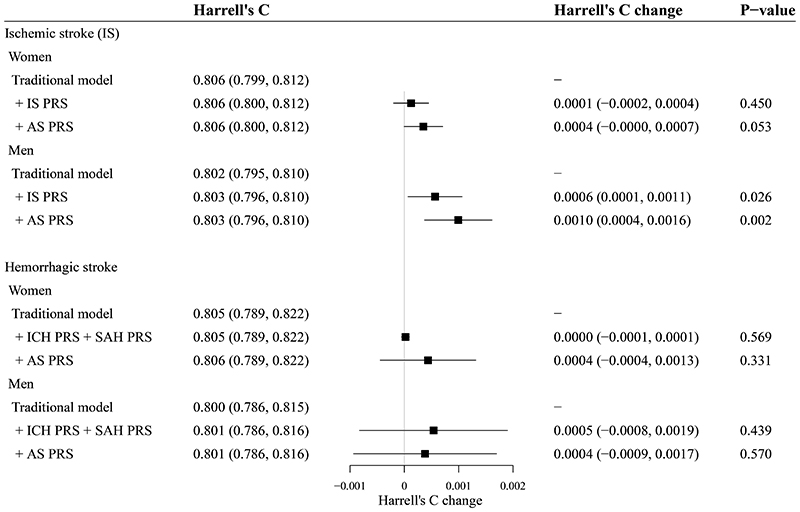
C statistics evaluating the performance of PRS CIs: Confidence intervals; CKB: China Kadoorie Biobank; CVD: Cardiovascular disease; ICD: International Classification of Disease; PRS: Polygenic risk score. The traditional risk prediction models (traditional models) were defined as sex-specific Cox models stratified by 10 study regions, with time on study as the time scale, including models for ischemic stroke (ICD-10: I63) and models for hemorrhagic stroke (ICD-10: I60-I62).[18] Predictors included in traditional models were the same as the “CKB-CVD models”, including age, systolic and diastolic blood pressure, use of anti-hypertensives, current daily smoking, self-reported diabetes, and waist circumference. Interactions between age and the other six predictors were also included. The 95% CIs of Harrell’s C and Harrell’s C changes were calculated by 100 bootstrap replications using the BCa method in Stata.

Based on the traditional models defined in this study, the addition of the PRS did notimprove or only slightly improve the discrimination performance of the models. For IS, the addition of PRS_AS_ increased Harrell’s C by 0.0010 in men (*P* = 0.002). For hemorrhagic stroke, the addition of PRSs did not influence Harrell’s C significantly (*P* > 0.05) (figure 3). The addition of the PRS offered little to no improvement in stroke risk stratification. For example, the categorical NRIs at the 10% high-risk threshold for ischemic and hemorrhagic stroke were all not significant in both sexes (*P* > 0.05) (supplemental table 7).

## Discussion

Based on the largest biobank in the Chinese population, only moderate associations were observed between PRSs and risks of stroke and its subtypes in this Chinese population, with an HR_SD_ of about 1.10. The addition of current PRSs offered little to no improvement in stroke risk prediction and risk stratification. We also found that the PRSs developed from GWAS summary statistics of IS were positively associated with the risk of ICH.

In the present study, the associations of PRSs with risks of stroke and its subtypes were moderate, suggesting a limited value for improving risk prediction over traditional risk factors. The HR_SD_ for PRS was usually greater than 1.20 in previous studies of the general population. A PRS for IS (PGS000039) that was developed with the metaGRS method and combined PRSs of 5 stroke subtypes and 14 stroke-related traits had an HR_SD_ of 1.26 (95% CI: 1.22-1.31) in the European population.[5] Another PRS for stroke (PGS002259) was also developed using the metaGRS method in a Chinese population, with the HR_SD_ for stroke being 1.28 (95% CI: 1.21-1.36).[10] However, these two PRSs showed much weaker associations with the risk of stroke or IS in the present study than in previous studies. Since both PRSs were developed using the elastic-net logistic regression, a machine learning approach, the potential overfitting may undermine their generalization performance.

The incidence rate of ICH is much higher in Chinese than in European populations. However, non-European populations are under-represented in GWAS, which serves as the basis for PRS development. The largest GWAS for ICH included only 3400 ICH cases, with most of them from European populations.[17] The present study attempted to develop PRS for ICH based on summary statistics from this GWAS. The weak associations observed in the present study are either explained by the difference in genetic background between ethnic groups or suggest that this GWAS may be underpowered. The stronger association estimate between PRS and HS risk reported in the previous study was likely due to the inclusion of PRSs for risk factors of HS (such as blood pressure) in the metaGRS method.[10] It is worth mentioning that, in the present study, the PRSs directly developed from GWAS summary statistics of IS were also positively associated with the risk of ICH. Although there are differences in etiology and risk factor profile between IS and ICH,[11–13] they might also have some partially shared etiological mechanisms like the cerebral small-vessel disease.[29]

This study has the following strengths. The large sample size and a large number of stroke events (including IS and ICH) enabled us to separate powerful training sets and the testing set and to conduct subgroup analyses. The loss to follow-up rate was less than 1% at an average follow-up period of over 12 years in CKB. The main subtypes of stroke (i.e., IS, ICH, and SAH) were well-classified, and the reporting and diagnostic accuracy of stroke events were high.[24] The genotyping and imputation of genetic data in this study were centrally conducted through a standard quality control process. Genetic variants with high reliability covered the whole genome well.

However, several limitations merit consideration. Firstly, we did not further consider the subtypes of IS (e.g., large-atherosclerotic stroke, cardioembolic stroke, and small vessel stroke) as over 75% of the incident IS events were coded as unspecified IS (ICD-10: I63.9), which precluded us from conducting more detailed analyses. Previous studies have suggested that there are differences in genetic loci of different IS subtypes.[14,30] Subsequent studies can explore whether distinguishing IS subtypes can further improve the predictive ability of PRS for IS. Secondly, compared with IS and ICH, the number of SAH events was relatively small. Therefore, it is difficult to exclude chance factors for the positive results observed in the present study. Further studies with more SAH events are warranted to examine our findings. Thirdly, the genetic variants with ambiguous SNP (i.e., A/T, C/G) and those that were not found in CKB or had low imputation quality scores were removed during the standard quality control process of PRSs. This might weaken the associations of previous PRSs with stroke and its subtypes. Fourthly, because information on blood lipids was not available for the current study population, we were unable to compare the impacts of blood lipids and PRS on traditional stroke risk prediction model improvement. However, the addition of blood lipids may enhance the traditional non-laboratory-based models, as previous studies have shown.[31,32] Therefore, adding PRS to a “lipid-enhanced model” might lead to a more minor improvement than what we have observed in the present study.

## Conclusions

In this Chinese population, the associations of optimal PRSs with risks of stroke and its subtypes were moderate, suggesting a limited value for improving risk prediction over traditional risk factors in the context of current GWAS underrepresenting the East Asian population. As GWAS of stroke and its subtypes progress among East Asians, further studies are warranted to assess whether new PRSs have considerable potential to translate into precision public health and population health benefits and, if so, to determine the appropriate context for their use.

## Supplementary Material

Checklists

Supplementary File 1

## Figures and Tables

**Table 1:** The optimal PRSs associated with risks of stroke and its subtypes in the training sets

Outcomes	Method	PRS source^a^	Number of variants	OR_SD_ (95% CI)	P-value	Note
Any stroke (N=7412 pairs)						
	Previous study	PGS002259	448	1.13 (1.09-1.16)	1.44×10^-11^	
	C+T	GCST005838 (*P*=1×10 6 r^2^=0)	38	1.11 (1.07-1.14)	1.90 ×10^9^	
	LDpred	GCST005838 (*ρ*=0.01, Ref=1KGP-EAS)	1,017,531	1.14 (1.10-1.18)	3.38×10^-14^	Optimal
Ischemic stroke (N=3844 pairs)						
	Previous study	PGS000039	1,563,569	1.07 (1.01-1.12)	0.012	
	C+T	GCST90018864 (*P*=0.02, r^2^=0.8)	32,158	1.18 (1.13-1.24)	3.55×10”	Optimal
	LDpred	GCST90018864 (*ρ*=0.01, Ref=1KGP-EUR)	1,017,672	1.17 (1.11-1.23)	1.46×10^9^	
Intracerebral hemorrhage (N=4296 pairs)						
	C+T	GCST90018870 (*P*=0.001, r^2^=0.2)	1326	1.09 (1.04-1.14)	1.37×10^-4^	
	LDpred	GCST90018870 (*ρ*=0.1, Ref=1KGP-EUR)	1,017,664	1.10 (1.05-1.15)	3.09×10’	Optimal
Subarachnoid hemorrhage (N=359 pairs)						
	C+T	GCST90018703 (*P*=0.4, r^2^=0)	7899	1.25 (1.06-1.47)	9.21×10 ^3^	Optimal
	LDpred	GCST90018923 (*ρ*=0.01, Ref=1KGP-EUR)	1,017,665	1.15 (0.98-1.35)	0.096	

Abbreviations: 1KGP, 1000 Genomes Project (Phase 3); CI, confidence interval; C+T, clumping & thresholding; EAS, East Asian; EUR, European; OR, odds ratio; PRS, polygenic risk score; Ref, reference population; SD, standard deviation. The current table only displays the optimal PRS obtained from different strategies (Previous study, C+T, and LDpred) for each disease outcome. The detailed results of all PRSs can be found in supplementary table 7.

a “PGS###” indicates the index in the PGS Catalog. “GCST###” indicates the index in the GWAS Catalog. The information in brackets is the parameter used for developing the PRS.

**Table 2:** Characteristics of the testing set

	Women	Men
**Number of participants**	43,170	28,980
**Baseline characteristics**		
Age, years	50.6 (42.5-58.3)	51.9 (43.2-60.3)
Rural areas	22,449 (52.0)	15,772 (54.4)
Array 1	5,948 (13.8)	4,503 (15.5)
Primary school and below	23,605 (54.7)	11,882 (41.0)
Daily smokers	915 (2.1)	16,317 (56.3)
Body mass index, kg/m^2^	23.6 (21.4-26.0)	23.3 (21.1-25.7)
Waist circumference, cm	78.0 (72.0-84.5)	81.5 (74.5-88.5)
Hypertension	14,062 (32.6)	10,653 (36.8)
Diabetes	2,477 (5.7)	1,553 (5.4)
Family history of stroke	7,619 (17.6)	5,075 (17.5)
**Follow-up**		
Follow-up time, years	12.6 (11.7-13.4)	12.4 (11.4-13.3)
Total person-years^a^	529,498	343,421
Incident events^b^		
Any stroke	4763 (11.0)	3751 (12.9)
Ischemic stroke	4254 (9.9)	3253 (11.2)
Intracerebral hemorrhage	600 (1.4)	593 (2.0)
Subarachnoid hemorrhage	87 (0.2)	45 (0.2)

Data are presented as n (%) or median (25–75th percentile) unless otherwise specified.

a Person-years were calculated as the time from the baseline date to the first of the following: death, loss to follow-up, or the global censoring date (December 31, 2018).

b Only the first event was counted.

## Data Availability

Details of how to access China Kadoorie Biobank data and details of the data release schedule are available from www.ckbiobank.org/site/Data+Access.

## Key Messages Box

**What is already known on this topic?**

- Polygenic risk scores (PRSs) might achieve risk stratification of stroke.
- Evidence from the East Asian population (including Chinese) is lacking.

**What this study adds?**

- The association strengths of current PRSs with risks of stroke and its subtypes were moderate in the Chinese population.
- PRS for ischemic stroke was positively associated with the risk of intracerebral hemorrhage.

**How this study might affect research, practice or policy?**

- In the Chinese population, current PRSs might have limited value for improving stroke risk prediction over traditional risk factors.
- Further studies are warranted to assess whether new PRSs based on larger GWAS or other developing methods have considerable potential to translate into population health benefits.

## Non-standard Abbreviations and Acronyms

CI: confidence interval
CKB: China Kadoorie Biobank
GWAS: genome-wide association study
HR: hazard ratio
ICH: intracerebral hemorrhage
IS: ischemic stroke
LD: linkage disequilibrium
OR: odds ratio
PRS: polygenic risk score
SAH: subarachnoid hemorrhage
SD: standard deviation
