# Supplementary material for: Associations of polygenic risk scores with risks of stroke and its subtypes in Chinese
Source: Stroke Vasc Neurol. Author manuscript; Available in PMC 2024 Aug 27. (PMC7616400; doi:10.1136/svn-2023-002428)
Supplement: Checklists [file EMS184994-supplement-Checklists.docx]

# Checklist 1: Polygenic Risk Score Reporting Standards (PRS-RS) Checklist

| **Manuscript Section** | **PRS-RS Item** | | **Page** |
| --- | --- | --- | --- |
| Introduction | Study Type | | 4 |
|  | Risk Model Purpose & Predicted Outcome | | 4 |
| Methods | Participants | Study Design & Recruitment | 5 |
|  |  | Demographic and Clinical Characteristics | 5 |
|  |  | Ancestry | 5 |
|  | Outcome of interest | | 7-8 |
|  | Non-Genetic Variables | | 6 |
|  | Genetic data | | 6-7 |
|  | Polygenic Risk Score Construction & Estimation | | 7 |
|  | Integrated Risk Model | Model Type | 9 |
|  |  | Model Fitting | 9 |
|  | Missing Data | | 6-7 |
|  | Statistical Methods | | 8-9 |
|  | Other Analyses | | N/A |
| Results | Participants | Demographic and Clinical Characteristics | 10-11 |
|  |  | Ancestry | 10 |
|  | PRS Distribution | | eFigure 2 |
|  | Risk Model Predictive Ability | | 11-12 |
|  | Risk Model Discrimination | | 11-12 |
|  | Risk Model Calibration | | N/A |
|  | Subgroup Analyses | | 11 |
| Discussion | Risk Model Interpretation | | 14 |
|  | Limitations | | 15 |
|  | Generalizability | | 13-14 |
|  | Risk Model Intended Uses | | N/A |
| Transparency and Reproducibility | Data Availability | | Title page |
|  | Funding | | Title page |

N/A=not applicable. This checklist was from the Supplemental Table 4 of Wand H, et al. *Nature* 2021.

# Checklist 2: STROBE Statement – cohort studies

|  | Item | Recommendation | Page |
| --- | --- | --- | --- |
| **Title and abstract** | 1 | (*a*) Indicate the study’s design with a commonly used term in the title or the abstract | 1 |
|  |  | (*b*) Provide in the abstract an informative and balanced summary of what was done and what was found | 1-2 |
| Introduction | | | |
| Background/rationale | 2 | Explain the scientific background and rationale for the investigation being reported | 4 |
| Objectives | 3 | State specific objectives, including any prespecified hypotheses | 4 |
| Methods | | | |
| Study design | 4 | Present key elements of study design early in the paper | 5-6 |
| Setting | 5 | Describe the setting, locations, and relevant dates, including periods of recruitment, exposure, follow-up, and data collection | 5 |
| Participants | 6 | (*a*) Give the eligibility criteria, and the sources and methods of selection of participants. Describe methods of follow-up | 5,7 |
|  |  | (*b*) For matched studies, give matching criteria and number of exposed and unexposed | N/A |
| Variables | 7 | Clearly define all outcomes, exposures, predictors, potential confounders, and effect modifiers. Give diagnostic criteria, if applicable | 5-9 |
| Data sources/ measurement | 8* | For each variable of interest, give sources of data and details of methods of assessment (measurement). Describe comparability of assessment methods if there is more than one group | N/A |
| Bias | 9 | Describe any efforts to address potential sources of bias | 8-9 |
| Study size | 10 | Explain how the study size was arrived at | 5 |
| Quantitative variables | 11 | Explain how quantitative variables were handled in the analyses. If applicable, describe which groupings were chosen and why | 8-9 |
| Statistical methods | 12 | (*a*) Describe all statistical methods, including those used to control for confounding | 8-9 |
|  |  | (*b*) Describe any methods used to examine subgroups and interactions | 9 |
|  |  | (*c*) Explain how missing data were addressed | N/A |
|  |  | (*d*) If applicable, explain how loss to follow-up was addressed | 7-8 |
|  |  | (*e*) Describe any sensitivity analyses | 11 |
| Results | | |  |
| Participants | 13* | (a) Report numbers of individuals at each stage of study—eg numbers potentially eligible, examined for eligibility, confirmed eligible, included in the study, completing follow-up, and analysed | 10-11 |
|  |  | (b) Give reasons for non-participation at each stage | N/A |
|  |  | (c) Consider use of a flow diagram | Fig. 1 |
| Descriptive data | 14* | (a) Give characteristics of study participants (eg demographic, clinical, social) and information on exposures and potential confounders | Table2 |
|  |  | (b) Indicate number of participants with missing data for each variable of interest | N/A |
|  |  | (c) Summarise follow-up time (eg, average and total amount) | 10 |
| Outcome data | 15* | Report numbers of outcome events or summary measures over time | 10-11 |

| Main results | 16 | (*a*) Give unadjusted estimates and, if applicable, confounder-adjusted estimates and their precision (eg, 95% confidence interval). Make clear which confounders were adjusted for and why they were included | eTable5 |
| --- | --- | --- | --- |
|  |  | (*b*) Report category boundaries when continuous variables were categorized | N/A |
|  |  | (*c*) If relevant, consider translating estimates of relative risk into absolute risk for a meaningful time period | N/A |
| Other analyses | 17 | Report other analyses done—eg analyses of subgroups and interactions, and sensitivity analyses | 11 |
| Discussion | | | |
| Key results | 18 | Summarise key results with reference to study objectives | 13 |
| Limitations | 19 | Discuss limitations of the study, taking into account sources of potential bias or imprecision. Discuss both direction and magnitude of any potential bias | 15 |
| Interpretation | 20 | Give a cautious overall interpretation of results considering objectives, limitations, multiplicity of analyses, results from similar studies, and other relevant evidence | 16 |
| Generalisability | 21 | Discuss the generalisability (external validity) of the study results | 13-14 |
| Other information | | | |
| Funding | 22 | Give the source of funding and the role of the funders for the present study and, if applicable, for the original study on which the present article is based | Title page |

*Give information separately for exposed and unexposed groups.
