# Supplementary material for: Associations of polygenic risk scores with risks of stroke and its subtypes in Chinese
Source: Stroke Vasc Neurol. Author manuscript; Available in PMC 2024 Aug 27. (PMC7616400; doi:10.1136/svn-2023-002428)
Supplement: Supplementary File 1 [file EMS184994-supplement-Supplementary_File_1.docx]

**Associations of polygenic risk scores with risks of stroke and its subtypes in Chinese**

**Supplemental Online Content**

[Members of the China Kadoorie Biobank collaborative group 2](#_Toc139525482)

[Supplemental methods 3](#_Toc139525483)

[Definition of the training sets 3](#_Toc139525484)

[Identification of previous PRS 3](#_Toc139525485)

[Identification of previous stroke-related GWAS 4](#_Toc139525486)

[Clumping & thresholding (C+T) method 5](#_Toc139525487)

[LDpred method 5](#_Toc139525488)

[Supplemental figure 1. High-quality variants in CKB 6](#_Toc139525489)

[Supplemental figure 2. Distributions and Correlation plots of the optimal PRSs for stroke and its subtypes in the testing set 7](#_Toc139525490)

[Supplemental figure 3. Associations of PRS with risk of ischemic stroke, stratified by different baseline characteristics 8](#_Toc139525491)

[Supplemental figure 4. Associations of PRS with risk of intracerebral hemorrhage, stratified by different baseline characteristics 9](#_Toc139525492)

[Supplemental table 1. The detailed process of case-control matching 10](#_Toc139525493)

[Supplemental table 2. Characteristics of the training sets 11](#_Toc139525494)

[Supplemental table 3. Quality control processes of PRS files from previous studies 12](#_Toc139525495)

[Supplemental table 4. Quality control processes of GWAS summary statistics files 13](#_Toc139525496)

[Supplemental table 5. Associations of different PRSs with risks of stroke and its subtypes in the training sets 15](#_Toc139525497)

[Supplemental table 6. Associations of PRSs with risks of stroke and subtypes after adjusting for systolic blood pressure, body mass index, and family history of stroke 17](#_Toc139525498)

[Supplemental table 7. Reclassification based on the continuous NRI and relative IDI 18](#_Toc139525499)

[References 19](#_Toc139525500)

# Members of the China Kadoorie Biobank collaborative group

**International Steering Committee:** Junshi Chen, Zhengming Chen (PI), Robert Clarke, Rory Collins, Yu Guo, Liming Li (PI), Jun Lv, Richard Peto, Robin Walters. **International Co-ordinating Centre, Oxford:** Daniel Avery, Derrick Bennett, Ruth Boxall, Sue Burgess, Ka Hung Chan, Yumei Chang, Yiping Chen, Zhengming Chen, Johnathan Clarke; Robert Clarke, Huaidong Du, Ahmed Edris Mohamed, Zammy Fairhurst-Hunter, Hannah Fry, Simon Gilbert, Alex Hacker, Mike Hill, Michael Holmes, Pek Kei Im, Andri Iona, Maria Kakkoura, Christiana Kartsonaki, Rene Kerosi, Kuang Lin, Mohsen Mazidi, Iona Millwood, Sam Morris, Qunhua Nie, Alfred Pozarickij, Paul Ryder, Saredo Said, Sam Sansome, Dan Schmidt, Paul Sherliker, Rajani Sohoni, Becky Stevens, Iain Turnbull, Robin Walters, Lin Wang, Neil Wright, Ling Yang, Xiaoming Yang, Pang Yao. **National Co-ordinating Centre, Beijing:** Yu Guo, Xiao Han, Can Hou, Jun Lv, Pei Pei, Chao Liu, Canqing Yu, Qingmei Xia. **10 Regional Co-ordinating Centres: Qingdao CDC:** Zengchang Pang, Ruqin Gao, Shanpeng Li, Haiping Duan, Shaojie Wang, Yongmei Liu, Ranran Du, Yajing Zang, Liang Cheng, Xiaocao Tian, Hua Zhang, Yaoming Zhai, Feng Ning, Xiaohui Sun, Feifei Li. **Licang CDC:** Silu Lv, Junzheng Wang, Wei Hou. **Heilongjiang Provincial CDC:** Wei Sun, Shichun Yan, Xiaoming Cui. **Nangang CDC:** Chi Wang, Zhenyuan Wu,Yanjie Li, Quan Kang. **Hainan Provincial CDC:** Huiming Luo, Tingting Ou. **Meilan CDC:** Xiangyang Zheng, Zhendong Guo, Shukuan Wu, Yilei Li, Huimei Li. **Jiangsu Provincial CDC:** Ming Wu, Yonglin Zhou, Jinyi Zhou, Ran Tao, Jie Yang, Jian Su. **Suzhou CDC:** Fang Liu, Jun Zhang, Yihe Hu, Yan Lu, Liangcai Ma, Aiyu Tang, Shuo Zhang, Jianrong Jin, Jingchao Liu. **Guangxi Provincial CDC:** Mei Lin, Zhenzhen Lu. **Liuzhou CDC:** Lifang Zhou, Changping Xie, Jian Lan,Tingping Zhu,Yun Liu, Liuping Wei, Liyuan Zhou, Ningyu Chen, Yulu Qin, Sisi Wang. **Sichuan Provincial CDC:** Xianping Wu, Ningmei Zhang, Xiaofang Chen, Xiaoyu Chang. **Pengzhou CDC:** Mingqiang Yuan, Xia Wu, Xiaofang Chen, Wei Jiang, Jiaqiu Liu, Qiang Sun. **Gansu Provincial CDC:** Faqing Chen, Xiaolan Ren, Caixia Dong. **Maiji CDC:** Hui Zhang, Enke Mao, Xiaoping Wang, Tao Wang, Xi zhang. **Henan Provincial CDC:** Kai Kang, Shixian Feng, Huizi Tian, Lei Fan. **Huixian CDC:** XiaoLin Li, Huarong Sun, Pan He, Xukui Zhang. **Zhejiang Provincial CDC:** Min Yu, Ruying Hu, Hao Wang. **Tongxiang CDC**: Xiaoyi Zhang, Yuan Cao, Kaixu Xie, Lingli Chen, Dun Shen. **Hunan Provincial CDC:** Xiaojun Li, Donghui Jin, Li Yin, Huilin Liu, Zhongxi Fu. **Liuyang CDC:** Xin Xu, Hao Zhang, Jianwei Chen,Yuan Peng, Libo Zhang, Chan Qu.

# Supplemental methods

## Definition of the training sets

Among the potential training set (n=22,191), every incident stroke event observed during follow-up was recorded. Only the first event was considered in the present analysis. All stroke events were sorted in order of date of birth ("potential case group"). The potential control group consisted of participants who did not have an incident stroke event during follow-up. In turn, each case was 1:1 matched with control for the study area, sex, and year of birth. The censored age of the control participant(s) should be larger than the age of the case. When multiple potential controls met the above criteria, one control was randomly selected. Each participant could only be selected as a control once. If no control was identified, we expanded the year of birth selection by ±1 year, ±2 years, and ±3 years. If the above procedure still failed to match a case with appropriate control, the case was excluded from the subsequent analysis.

Finally, 7412 (74.3%) of 9977 incident stroke cases were successfully matched with controls. Following a similar procedure, 3844 (74.6%) of 5154 incident ischemic stroke cases, 4296 (95.2%) of 4514 incident intracerebral hemorrhage cases, and 359 (98.4%) of 365 incident subarachnoid hemorrhage cases were successfully matched with controls (**figure 1, supplemental table 1, supplemental table 2**).

## Identification of previous PRS

We systematically searched the PGS Catalog,^[1]^ PubMed, and Embase to obtain stroke-related PRS directly from previous studies (Date of searching: 2022-08-06). The detailed search strategies were below:

PGS Catalog

stroke

PubMed

#1: stroke[TI] OR "cerebral infarct*"[TI] OR "intracerebral hemorrhage"[TI] OR "intracerebral haemorrhage"[TI] OR "subarachnoid hemorrhage"[TI] OR "subarachnoid haemorrhage"[TI]

#2: "genetic risk*"[TI] OR "genetic tool*"[TI] OR "polygenic risk*"[TI] OR "polygenic score*"[TI] OR "genomic risk*"[TI]

#3: Review[PT] OR Comment[PT] OR Editorial[PT] OR "Published Erratum"[PT]

#4: "genetic risk factor*"[TI] OR "reply"[TI]

Final: #1 AND #2 NOT (#3 OR #4)

Embase

#1: 'stroke':ti OR 'cerebral infarction':ti OR 'intracerebral hemorrhage':ti OR 'intracerebral haemorrhage':ti OR 'subarachnoid hemorrhage':ti OR 'subarachnoid haemorrhage':ti

#2: 'genetic risk':ti OR 'genetic tool$':ti OR 'polygenic risk':ti OR 'polygenic score$':ti OR 'genomic risk':ti

#3: [article]/lim AND [english]/lim AND [embase]/lim

#4: 'genetic risk factor$':ti

Final: #1 AND #2 AND #3 NOT #4

The inclusion criteria of PRS in the current study were as follows:

- Newly developed.
- The PRS should integrate the information of multiple genetic variants across the whole genome and calculate individual genetic risk by weighted sum.
- The target trait of PRS should be stroke or subtypes of stroke, including ischemic stroke, intracerebral hemorrhage, and subarachnoid hemorrhage.
- The primary purpose of the original study was to examine the strength of association between PRS and stroke or subtypes of stroke, or to evaluate the effect of PRS on improving a risk prediction model for stroke or subtypes of stroke.

The exclusion criteria of PRS were as follows:

- The base data of PRS did not include GWAS of stroke or subtypes of stroke. For example, the PRS developed using blood-pressure-related genetic variants.
- Variants in PRS were selected only based on genome-wide significant variants of stroke.
- The training set of PRS was a population with a certain disease (such as individuals with cardiometabolic disease or atrial fibrillation, etc.).
- The information used to construct a PRS (i.e., chromosome, position, effect allele, weight, etc.) was not publicly available from the PGS Catalog website or the supplemental files of the original study.

Following the above search strategy and inclusion and exclusion criteria, four previously reported PRSs were identified. Standard quality control for genetic variants was conducted before subsequent analysis (**Supplemental table 3**).

## Identification of previous stroke-related GWAS

We systematically searched previous studies using *gwasfilter*, a customized R script that can efficiently and accurately filter GWASs from the GWAS Catalog Website.^[2]^ GWASs can be filtered based on "whether the GWAS has been replicated", "sample size", "ethnicity of the study population", and other conditions. The source code of this R script is available on GitHub (<https://github.com/lab319/gwas_filter>). (Date of searching: 2022-08-06)

The detailed search strategies were below:

## Step 1: Load this script

source("gwasfilter.R")

## Step 2: Download the latest database from the GWAS Catalog

get_gwasdata()

## Step 3: Determine the filtering strategies for each trait one by one

# any stroke (EFO_0000712)

get_efo(trait="stroke")

obtain_trait(efoindex=1, append=F)

store_trait(traitindex=c(1))

gwasfilter(association=T)

# ischemic stroke (HP_0002140)

get_efo(trait="ischemic stroke")

obtain_trait(efoindex=1, append=T)

store_trait(traitindex=c(1,3,8,17))

# intracerebral hemorrhage (EFO_0005669)

get_efo(trait="intracerebral hemorrhage")

obtain_trait(efoindex=1, append=T)

store_trait(traitindex=c(1,2,10))

# subarachnoid hemorrhage (EFO_0000713)

get_efo(trait="subarachnoid hemorrhage")

obtain_trait(efoindex=1, append=T)

store_trait(traitindex=c(1:6))

## Step 4: Export the study list

gwasfilter(association=F)

Finally, based on ethnicity, sample size, and accessibility of the summary statistics file (SSF), we included 1 stroke SSF, 2 IS SSFs, 2 ICH SSFs, and 2 SAH SSFs from two large-scale GWASs (**supplemental table 4**).^[3,4]^

## Clumping & thresholding (C+T) method

This approach involves taking the estimated single nucleotide polymorphism (SNP) effects from the largest available GWAS as the SNP weights. In the current study, a grid search strategy was used to construct multiple sets of PRS: we applied the r^2^ threshold as 0 (=no pruning), 0.2, 0.4, 0.6, and 0.8, and the P-value threshold from 5×10^-8^ to 1 (40 values in total). For each r^2^ threshold, we used PLINK 1.9 ^[5]^ to prune variants separately for the 22 autosomes (--clump-kb 250). The threshold on P-value was not applied during linkage disequilibrium (LD) pruning (--clump-p1 1). The reference panel used for LD pruning was 1595 unrelated participants from CKB. We then applied different thresholds to the P-value for associations from the original GWAS. The PRS was computed by a weighted sum of the SNP dosages. After the above process, a GWAS summary statistics file could produce 5×40=200 PRSs with different r^2^ thresholds and different P-value thresholds.

## LDpred method

This Bayesian approach calculates a posterior mean effect for each variant based on a prior and subsequent shrinkage based on the extent to which this variant is correlated with similarly associated variants in the reference population.^[6]^ Three steps are involved to develop PRS by LDpred (v1.0.10): (1) coordination of SNPs; (2) calculation of SNP posterior effects; (3) calculation of PRS. The variants were restricted to HapMap3 SNPs in the current analysis. Two parameters were required to run LDpred. The first parameter is the LD radius, i.e., the number of SNPs that we adjust for on each side of a given SNP. We used M/3000, the default value recommended by the software, where M is the total number of SNPs used in the analysis. This corresponds to a 2 Mb LD window on average in the genome. The second parameter is the fraction *p* of non-zero effects in the prior. A range of *p* values recommended by the software were used: 1, 0.3, 0.1, 0.03, 0.01, 0.003, and 0.001. In addition, the LD reference panel was required to compute the correlations between genetic variants. East Asians (n=504) and Europeans (n=503) in 1000 Genomes Project Phase 3 were used as LD reference panels, respectively.

# Supplemental figure 1. High-quality variants in CKB


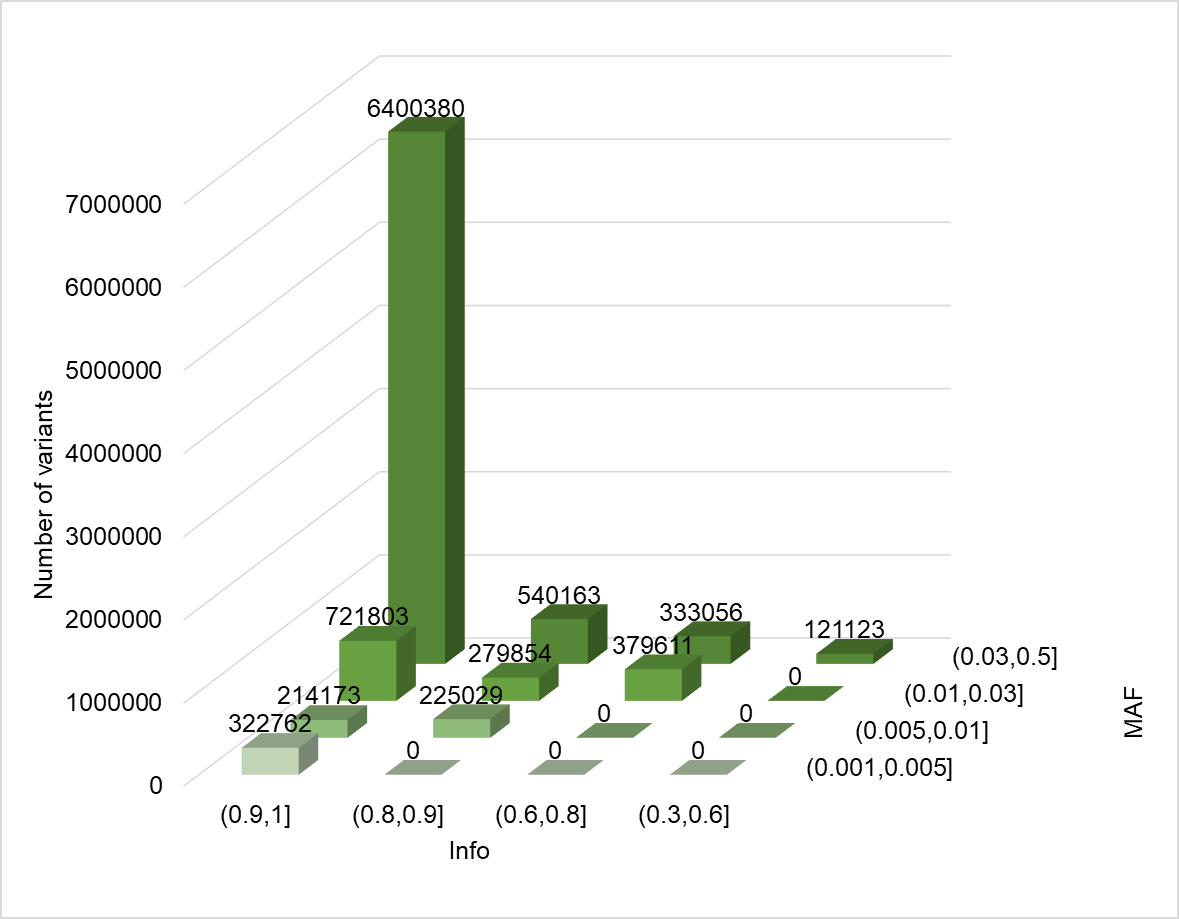


Abbreviations: Info, imputation quality score; MAF, minor allele frequency.

High-quality variants were defined as: (1) Info>0.3 & MAF>0.03; or (2) Info>0.6 & MAF>0.01; or (3) Info>0.8 & MAF>0.005; or (4) Info>0.9 & MAF>0.001.

# Supplemental figure 2. Distributions and Correlation plots of the optimal PRSs for stroke and its subtypes in the testing set


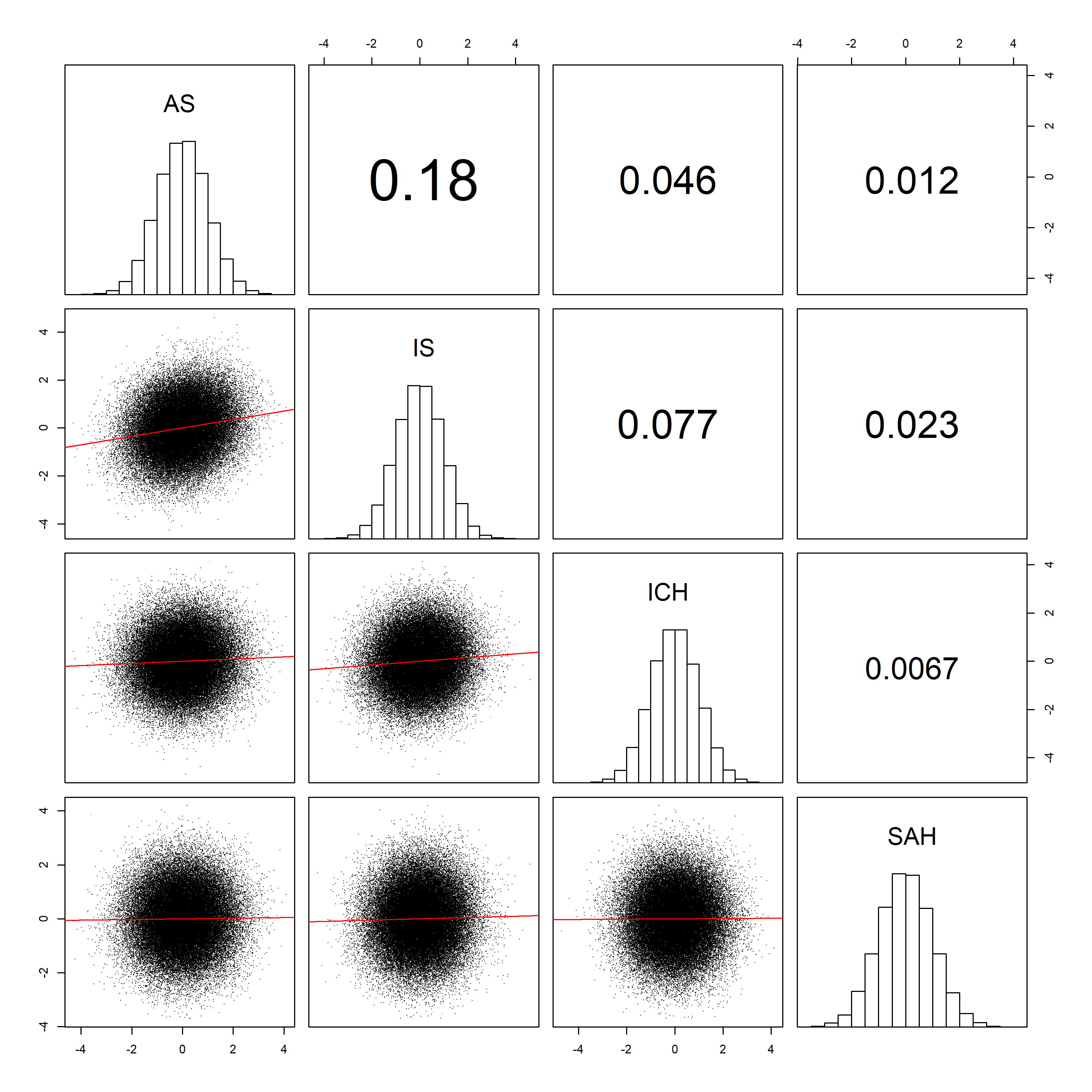


Abbreviations: AS, any stroke; ICH, intracerebral hemorrhage; IS, ischemic stroke; PRS, polygenic risk score; SAH, subarachnoid hemorrhage.

The PRSs reported here are the optimal PRSs for stroke and its subtypes in the training sets (see **table 1** for details). All PRSs were standardized in the testing set (n=72,150) before plotting. The number in the upper-right square of the plot represents the Pearson correlation coefficient. The red line in the lower-left square represents the regression line.

# Supplemental figure 3. Associations of PRS with risk of ischemic stroke, stratified by different baseline characteristics


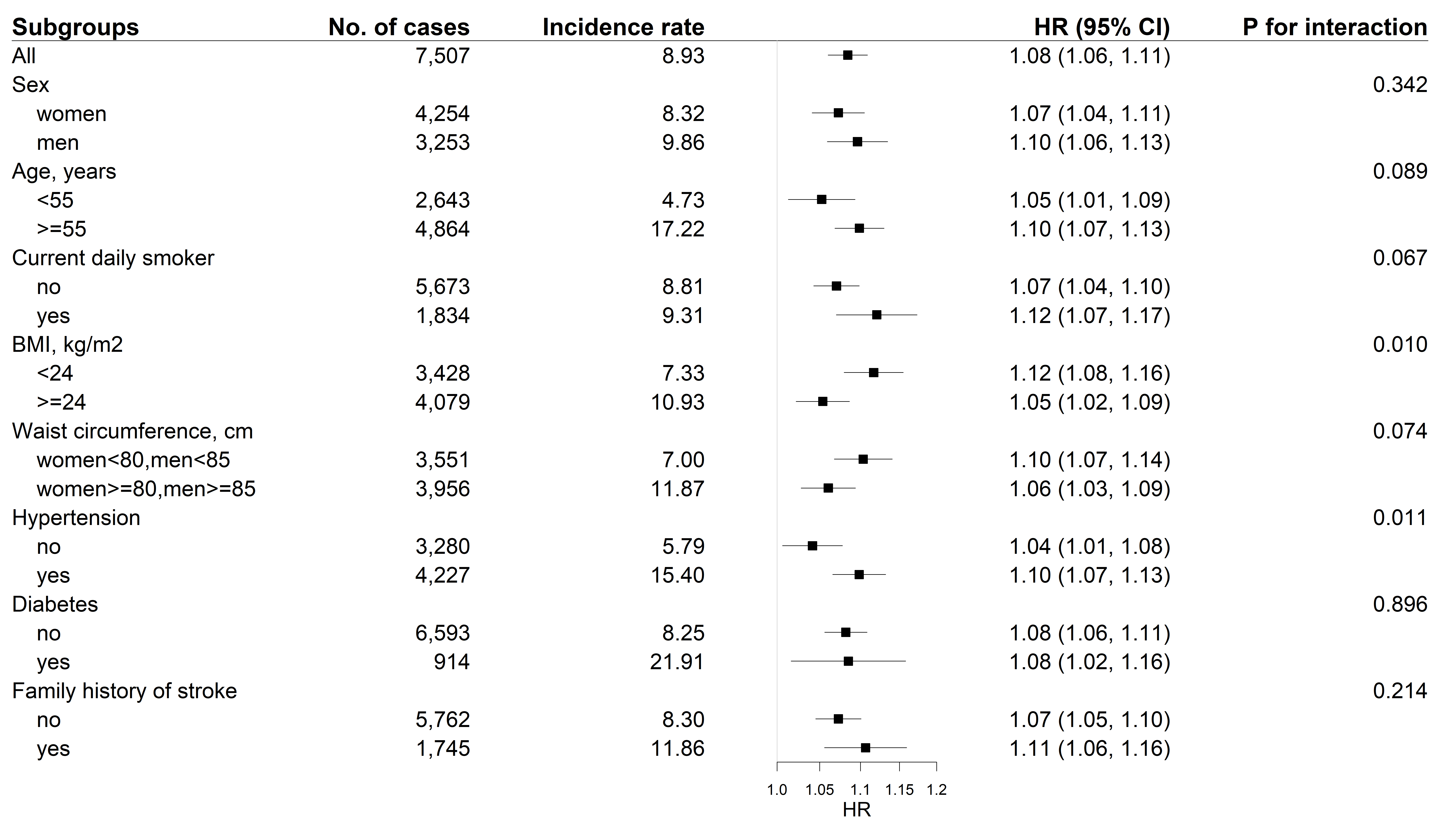


The PRS reported here is the optimal PRS for ischemic stroke (see **table 1**), which was standardized (zero mean, unit standard deviation) in the testing set. The incidence rate is reported in unit per 1000 person-years. The Cox models were stratified by sex and ten study regions and adjusted simultaneously for the top 10 principal components of ancestry and array versions, with age as the time scale. The tests for multiplicative interaction were performed using likelihood ratio tests by comparing models with and without cross-product terms. One participant had missing value of body mass index (BMI) and was excluded when stratified by BMI.

# Supplemental figure 4. Associations of PRS with risk of intracerebral hemorrhage, stratified by different baseline characteristics


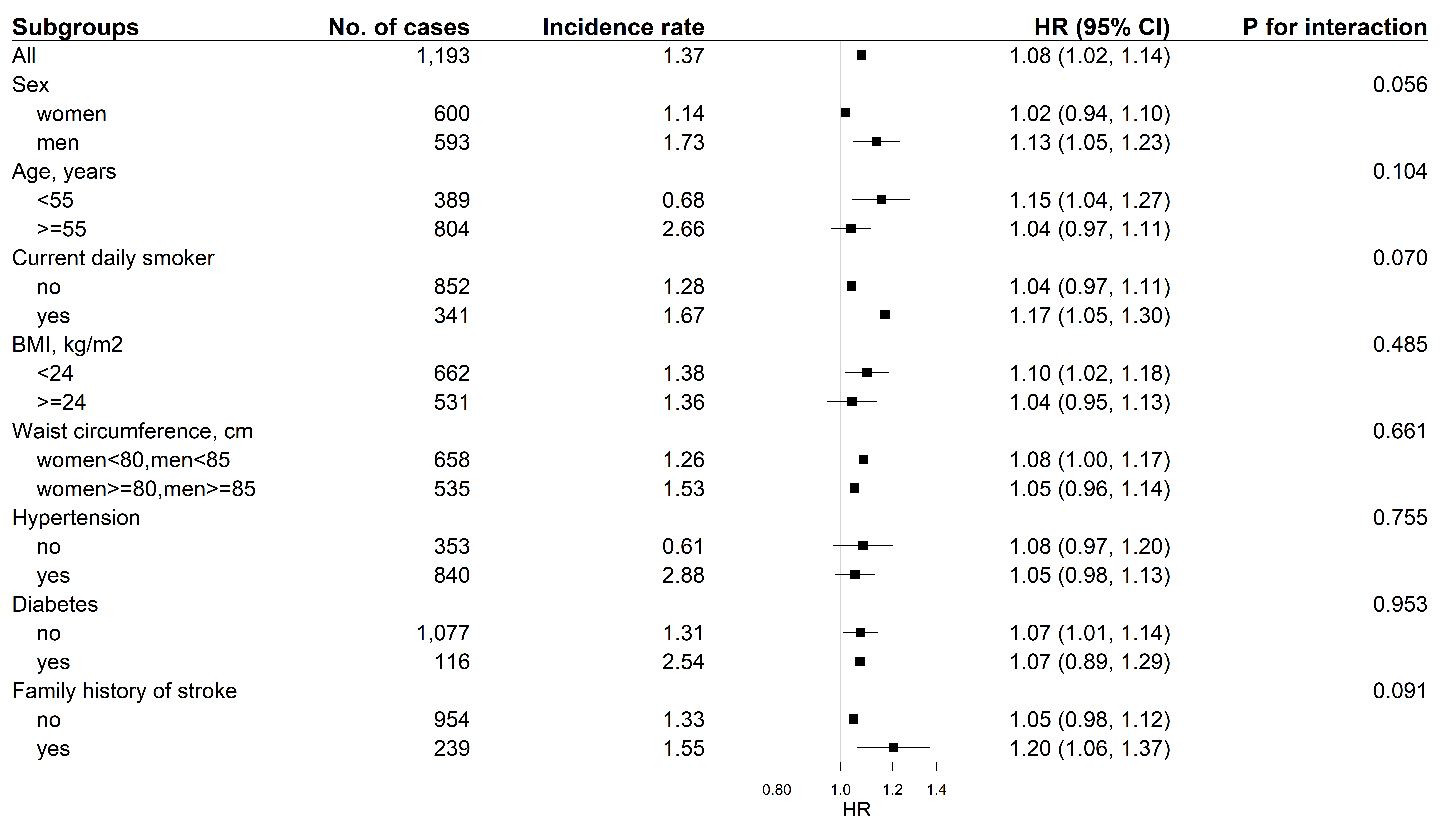


The PRS reported here is the optimal PRS for intracerebral hemorrhage (see **table 1**), which was standardized (zero mean, unit standard deviation) in the testing set. The incidence rate is reported in unit per 1000 person-years. The Cox models were stratified by sex and ten study regions and adjusted simultaneously for the top 10 principal components of ancestry and array versions, with age as the time scale. The tests for multiplicative interaction were performed using likelihood ratio tests by comparing models with and without cross-product terms. One participant had missing value of body mass index (BMI) and was excluded when stratified by BMI.

# Supplemental table 1. The detailed process of case-control matching

| Steps | Difference between the year of birth (control - case) | Number of matched cases | | | |
| --- | --- | --- | --- | --- | --- |
|  |  | AS | IS | ICH | SAH |
| 1 | 0 | 6825 | 3458 | 3978 | 324 |
| 2 | -1 | 267 | 199 | 159 | 20 |
| 3 | 1 | 104 | 59 | 59 | 4 |
| 4 | -2 | 115 | 65 | 45 | 7 |
| 5 | 2 | 30 | 20 | 20 | 1 |
| 6 | -3 | 58 | 34 | 27 | 1 |
| 7 | 3 | 13 | 9 | 8 | 2 |
| Summing | | 7412 | 3844 | 4296 | 359 |

Abbreviations: AS, any stroke; ICH, intracerebral hemorrhage; IS, ischemic stroke; SAH, subarachnoid hemorrhage.

# Supplemental table 2. Characteristics of the training sets

|  | **Case** | **Control** |
| --- | --- | --- |
| **The training set for any stroke** |  |  |
| Number of participants | 7412 | 7412 |
| Array 1 | 6133 (82.7) | 6902 (93.1) |
| Rural area | 5392 (72.7) | 5392 (72.7) |
| Men | 3848 (51.9) | 3848 (51.9) |
| Censored age, years | 71.0 (63.5-77.9) | 65.3 (57.0-72.0) |
| **The training set for ischemic stroke** |  |  |
| Number of participants | 3844 | 3844 |
| Array 1 | 3010 (78.3) | 3681 (95.8) |
| Rural area | 2369 (61.6) | 2369 (61.6) |
| Men | 1941 (50.5) | 1941 (50.5) |
| Censored age, years | 69.6 (62.3-76.5) | 64.1 (56.1-70.6) |
| **The training set for intracerebral hemorrhage** |  |  |
| Number of participants | 4296 | 4296 |
| Array 1 | 3606 (83.9) | 3887 (90.5) |
| Rural area | 3348 (77.9) | 3348 (77.9) |
| Men | 2294 (53.4) | 2294 (53.4) |
| Censored age, years | 72.1 (64.6-78.9) | 65.9 (57.7-73.0) |
| **The training set for subarachnoid hemorrhage** |  |  |
| Number of participants | 359 | 359 |
| Array 1 | 284 (79.1) | 332 (92.5) |
| Rural area | 229 (63.8) | 229 (63.8) |
| Men | 138 (38.4) | 138 (38.4) |
| Censored age, years | 67.9 (60.8-75.5) | 61.0 (53.8-69.2) |

Data are presented as n (%) or median (25–75th percentile) unless otherwise specified.

# Supplemental table 3. Quality control processes of PRS files from previous studies

| **Index** | **PRS ID** | **Outcomes** | **Development methods** | **First Author**  **(Publication year)** | **The original** **number of variants** | **Matched with CKB** | **Non-ambiguous** | **Non-Ins/Del** | **Info ≥ 0.8 in CKB** | **MAF ≥ 1% in CKB** | **P_HWE_ ≥ 1×10^-6^ in CKB** |
| --- | --- | --- | --- | --- | --- | --- | --- | --- | --- | --- | --- |
| 1 | PGS000038 | Stroke | C+T | Rutten-Jacobs LC  (2018) | 90 | 73 | 67 | 67 | 67 | 59 | 59 |
| 2 | PGS000039 | Ischemic stroke | metaGRS | Abraham G  (2019) | 3,225,583 | 2,353,410 | 2,012,084 | 2,012,084 | 1,655,235 | 1,592,365 | 1,563,569 |
| 3 | PGS002259 | Stroke | metaGRS | Lu X  (2021) | 534 | 532 | 473 | 473 | 467 | 456 | 448 |
| 4 | GRS324 | Stroke | metaGRS | Ibrahim-Verbaas CA  (2014) | 324 | 321 | 278 | 278 | 278 | 246 | 241 |

Abbreviations: C+T, clumping & thresholding; CKB, China Kadoorie Biobank; HWE, Hardy-Weinberg Equilibrium; Info, imputation quality score; Ins/Del, insertion/deletion; MAF, minor allele frequency; PRS, polygenic risk score.

# Supplemental table 4. Quality control processes of GWAS summary statistics files

| **Items** | **Source 1** | **Source 2** | **Source 3** | **Source 4** | **Source 5** | **Source 6** | **Source 7** |
| --- | --- | --- | --- | --- | --- | --- | --- |
| ID in GWAS Catalog | GCST005838 | GCST90018703 | GCST90018923 | GCST90018650 | GCST90018870 | GCST90018644 | GCST90018864 |
| Outcomes | Stroke | Subarachnoid hemorrhage | Subarachnoid hemorrhage | Intracerebral hemorrhage | Intracerebral hemorrhage | Ischemic stroke | Ischemic stroke |
| Sample size | 67,162 multi-ancestry cases / 454,450 multi-ancestry controls | 1,203 EAS cases / 152,022 EAS controls | 1,693 EUR cases /471,562 EUR controls + 1,203 EAS cases / 152,022 EAS controls | 1,456 EAS cases /152,022 EAS controls | 1,935 EUR cases / 471,578 EUR controls + 1,456 EAS cases / 152,022 EAS controls | 22,664 EAS cases / 152,022 EAS controls | 11,929 EUR cases / 472,192 EUR controls +  22,664 EAS cases / 152,022 EAS controls |
| First Author  (Publication year) | Malik R  (2018) | Sakaue S  (2021) | Sakaue S  (2021) | Sakaue S  (2021) | Sakaue S  (2021) | Sakaue S  (2021) | Sakaue S  (2021) |
| The original number of variants | 7,675,830 | 13,425,781 | 25,841,499 | 13,425,819 | 25,841,532 | 13,429,439 | 25,844,498 |
| Passed initial quality control | 7,627,850 ^a^ | 13,130,774 ^b^ | 24,878,293 ^c^ | 13,130,813 ^d^ | 24,878,319 ^e^ | 13,134,427 ^f^ | 24,881,216 ^g^ |
| MAF ≥ 1% in GWAS | 7,627,850 ^h^ | 7,440,434 | 11,513,781 | 7,440,398 | 11,513,800 | 7,440,112 | 11,515,909 |
| Matched with CKB | 6,475,513 | 7,299,168 | 9,261,503 | 7,299,120 | 9,261,488 | 7,298,797 | 9,264,734 |
| Non-ambiguous | 5,478,155 | 6,257,327 | 7,965,690 | 6,257,306 | 7,965,668 | 6,257,016 | 7,968,457 |
| Non-Ins/Del | 5,477,165 | 5,778,284 | 7,025,494 | 5,778,261 | 7,025,479 | 5,777,991 | 7,028,074 |
| Info ≥ 0.8 in CKB | 4,869,469 | 5,207,701 | 5,820,101 | 5,207,708 | 5,820,133 | 5,207,526 | 5,822,588 |
| MAF ≥ 1% in CKB | 4,612,892 | 5,087,906 | 5,475,815 | 5,087,897 | 5,475,832 | 5,087,676 | 5,478,538 |
| P_HWE_ ≥ 1×10^-6^ in CKB | 4,557,514 | 5,028,354 | 5,402,594 | 5,028,344 | 5,402,611 | 5,028,124 | 5,405,310 |
| In HapMap3 | 1,022,347 | 993,361 | 1,029,250 | 993,366 | 1,027,999 | 993,337 | 1,028,074 |
| MAF ≥ 1% in 1KGP_EAS_ | 1,017,531 | 991,773 | 1,024,440 | 991,780 | 1,023,197 | 991,768 | 1,023,272 |

Abbreviations: 1KGP, 1000 Genomes Project (Phase 3); CKB, China Kadoorie Biobank; EAS, East Asian; EUR, European; GWAS, genome-wide association study; HapMap3, the International HapMap Project Phase 3; HWE, Hardy-Weinberg Equilibrium; Info, imputation quality score; Ins/Del, insertion/deletion; MAF, minor allele frequency;

^a^ We excluded 47,087 variants whose chromosomes or positions were not available and 893 variants that were in the same position as other variants.

^b^ We excluded 295,007 variants on chromosome X.

^c^ We excluded 798,531 variants on chromosome X and 164,675 variants that were at the same position as other variants.

^d^ We excluded 295,006 variants on chromosome X.

^e^ We excluded 798,538 variants on chromosome X and 164,675 variants that were at the same position as other variants.

^f^ We excluded 295,012 variants on chromosome X.

^g^ We excluded 798,593 variants on chromosome X and 164,689 variants that were at the same position as other variants.

^h^ MAF was not available in the summary statistics file.

# Supplemental table 5. Associations of different PRSs with risks of stroke and its subtypes in the training sets

| **Outcomes** | **Method** | **PRS source ^a^** | **Parameter used for developing the PRS in the present study** | **Number of variants** | **OR_SD_ (95% CI)** | **P-value** | **Note** |
| --- | --- | --- | --- | --- | --- | --- | --- |
| Any stroke |  |  |  |  |  |  |  |
|  | Previous study | PGS000038 | — | 59 | 1.058 (1.024, 1.094) | 7.66E-04 |  |
|  | Previous study | PGS002259 | — | 448 | 1.125 (1.088, 1.165) | 1.44E-11 |  |
|  | Previous study | GRS324 | — | 241 | 1.015 (0.982, 1.050) | 3.71E-01 |  |
|  | C + T | GCST005838 | *P*=1E-06, r^2^=0 | 38 | 1.107 (1.071, 1.145) | 1.90E-09 |  |
|  | **LDpred** | **GCST005838** | **ρ=0.01, Ref=1KGP-EAS** | **1,017,531** | **1.138 (1.101, 1.177)** | **3.38E-14** | **Optimal** |
|  | LDpred | GCST005838 | ρ=0.01, Ref=1KGP-EUR | 1,017,496 | 1.131 (1.094, 1.170) | 5.96E-13 |  |
| Ischemic stroke |  |  |  |  |  |  |  |
|  | Previous study | PGS000039 | — | 1,563,569 | 1.065 (1.014, 1.119) | 1.16E-02 |  |
|  | C + T | GCST90018644 | *P*=0.07, r^2^=0.8 | 74,670 | 1.131 (1.077, 1.188) | 7.19E-07 |  |
|  | **C + T** | **GCST90018864** | ***P*=0.02, r^2^=0.8** | **32,158** | **1.183 (1.126, 1.244)** | **3.55E-11** | **Optimal** |
|  | LDpred | GCST90018644 | ρ=0.001, Ref=1KGP-EAS | 991,768 | 1.116 (1.063, 1.172) | 1.12E-05 |  |
|  | LDpred | GCST90018644 | ρ=0.03, Ref=1KGP-EUR | 982,412 | 1.102 (1.050, 1.157) | 8.47E-05 |  |
|  | LDpred | GCST90018864 | ρ=0.01, Ref=1KGP-EAS | 1,023,272 | 1.162 (1.106, 1.220) | 1.80E-09 |  |
|  | LDpred | GCST90018864 | ρ=0.01, Ref=1KGP-EUR | 1,017,672 | 1.166 (1.110, 1.226) | 1.46E-09 |  |
| Intracerebral hemorrhage |  |  |  |  |  |  |  |
|  | C + T | GCST90018650 | *P*=0.2, r^2^=0.8 | 192,079 | 1.082 (1.037, 1.130) | 3.20E-04 |  |
|  | C + T | GCST90018870 | *P*=0.001, r^2^=0.2 | 1,326 | 1.088 (1.042, 1.136) | 1.37E-04 |  |
|  | LDpred | GCST90018650 | ρ=0.003, Ref=1KGP-EAS | 991,780 | 1.066 (1.020, 1.114) | 4.17E-03 |  |
|  | LDpred | GCST90018650 | ρ=0.01, Ref=1KGP-EUR | 982,436 | 1.073 (1.028, 1.121) | 1.44E-03 |  |
|  | LDpred | GCST90018870 | ρ=0.003, Ref=1KGP-EAS | 1,023,197 | 1.087 (1.041, 1.135) | 1.61E-04 |  |
|  | **LDpred** | **GCST90018870** | **ρ=0.1, Ref=1KGP-EUR** | **1,017,664** | **1.097 (1.050, 1.146)** | **3.09E-05** | **Optimal** |
| Subarachnoid hemorrhage |  |  |  |  |  |  |  |
|  | **C + T** | **GCST90018703** | ***P*=0.4, r^2^=0** | **7,899** | **1.248 (1.056, 1.475)** | **9.21E-03** | **Optimal** |
|  | C + T | GCST90018923 | *P*=0.0005, r^2^=0.8 | 889 | 1.246 (1.064, 1.458) | 6.20E-03 |  |
|  | LDpred | GCST90018703 | ρ=0.001, Ref=1KGP-EAS | 991,773 | 1.082 (0.933, 1.255) | 2.99E-01 |  |
|  | LDpred | GCST90018703 | ρ=0.001, Ref=1KGP-EUR | 982,431 | 1.126 (0.967, 1.311) | 1.26E-01 |  |
|  | LDpred | GCST90018923 | ρ=0.001, Ref=1KGP-EAS | 1,024,440 | 1.129 (0.962, 1.325) | 1.37E-01 |  |
|  | LDpred | GCST90018923 | ρ=0.01, Ref=1KGP-EUR | 1,017,665 | 1.147 (0.976, 1.348) | 9.61E-02 |  |

Abbreviations: 1KGP, 1000 Genomes Project (Phase 3); CI, confidence interval; C+T, clumping & thresholding; EAS, East Asian; EUR, European; OR, odds ratio; PRS, polygenic risk score; Ref, reference population; SD, standard deviation.

^a^ "PGS###" indicates the index in the PGS Catalog. "GCST###" indicates the index in the GWAS Catalog.

# Supplemental table 6. Associations of PRSs with risks of stroke and subtypes after adjusting for systolic blood pressure, body mass index, and family history of stroke

| **Outcomes** | **PRS** | **Model 1** | **Model 2** | **Model 3** |
| --- | --- | --- | --- | --- |
| Any stroke |  |  |  |  |
|  | PRS_AS_ | **1.10 (1.07, 1.12)** | **1.10 (1.07, 1.12)** | **1.08 (1.06, 1.10)** |
|  | PRS_IS_ | **1.08 (1.06, 1.11)** | **1.08 (1.06, 1.11)** | **1.07 (1.05, 1.09)** |
|  | PRS_ICH_ | 1.02 (1.00, 1.04) | 1.02 (1.00, 1.04) | 1.01 (0.99, 1.03) |
|  | PRS_SAH_ | 1.00 (0.98, 1.02) | 1.00 (0.98, 1.03) | 1.00 (0.98, 1.02) |
| Ischemic stroke |  |  |  |  |
|  | PRS_AS_ | **1.10 (1.07, 1.12)** | **1.10 (1.07, 1.12)** | **1.08 (1.06, 1.11)** |
|  | PRS_IS_ | **1.08 (1.06, 1.11)** | **1.08 (1.06, 1.11)** | **1.07 (1.04, 1.09)** |
|  | PRS_ICH_ | 1.02 (0.99, 1.04) | 1.02 (0.99, 1.04) | 1.01 (0.99, 1.03) |
|  | PRS_SAH_ | 1.00 (0.98, 1.03) | 1.00 (0.98, 1.03) | 1.00 (0.98, 1.02) |
| Intracerebral hemorrhage |  |  |  |  |
|  | PRS_AS_ | **1.13 (1.07, 1.19)** | **1.13 (1.07, 1.20)** | **1.09 (1.03, 1.16)** |
|  | PRS_IS_ | **1.09 (1.03, 1.15)** | **1.09 (1.03, 1.15)** | **1.06 (1.00, 1.12) ^a^** |
|  | PRS_ICH_ | **1.08 (1.02, 1.14)** | **1.07 (1.01, 1.14)** | **1.07 (1.01, 1.13)** |
|  | PRS_SAH_ | 1.02 (0.96, 1.08) | 1.02 (0.96, 1.08) | 1.02 (0.96, 1.08) |
| Subarachnoid hemorrhage |  |  |  |  |
|  | PRS_AS_ | 1.10 (0.93, 1.30) | 1.10 (0.93, 1.31) | 1.07 (0.90, 1.27) |
|  | PRS_IS_ | 1.02 (0.86, 1.21) | 1.03 (0.86, 1.22) | 1.01 (0.85, 1.20) |
|  | PRS_ICH_ | 1.07 (0.90, 1.27) | 1.07 (0.90, 1.27) | 1.07 (0.90, 1.27) |
|  | PRS_SAH_ | 0.97 (0.81, 1.15) | 0.97 (0.82, 1.15) | 0.96 (0.81, 1.14) |

Abbreviations: AS, any stroke; ICH, intracerebral hemorrhage; IS, ischemic stroke; PRS, polygenic risk score; SAH, subarachnoid hemorrhage.

Model 1 was stratified by sex and ten study regions, with age as the time scale. Model 2 was further adjusted for the top 10 principal components of ancestry and array versions. Model 3 was further adjusted for systolic blood pressure, body mass index, and family history of stroke.

^a^ *P*<0.05.

# Supplemental table 7. Reclassification based on the continuous NRI and relative IDI

|  | categorical NRI ^a^ | continuous NRI | relative IDI ^b^, % |
| --- | --- | --- | --- |
| Ischemic stroke |  |  |  |
| Women |  |  |  |
| cases | 0.001 (-0.005, 0.006) | 0.039 (-0.003, 0.080) | – |
| non-cases | 0.001 (-0.000, 0.002) | **0.034 (0.024, 0.044)** | – |
| total | 0.001 (-0.004, 0.007) | **0.073 (0.030, 0.115)** | **0.5 (0.2, 0.9)** |
| Men |  |  |  |
| cases | 0.004 (-0.003, 0.012) | 0.035 (-0.007, 0.077) | – |
| non-cases | -0.001 (-0.003, 0.000) | **0.040 (0.029, 0.052)** | – |
| total | 0.003 (-0.004, 0.011) | **0.075 (0.031, 0.120)** | 0.4 (-0.1, 0.9) |
| Hemorrhagic stroke |  |  |  |
| Women |  |  |  |
| cases | -0.008 (-0.017, 0.001) | 0.007 (-0.086, 0.099) | – |
| non-cases | -0.000 (-0.000, 0.000) | **0.021 (0.011, 0.031)** | – |
| total | -0.008 (-0.017, 0.001) | 0.028 (-0.065, 0.121) | -0.2 (-1.1, 0.7) |
| Men |  |  |  |
| cases | 0.008 (-0.008, 0.024) | 0.092 (-0.000, 0.184) |  |
| non-cases | 0.000 (-0.000, 0.001) | **0.039 (0.028, 0.049)** |  |
| total | 0.008 (-0.008, 0.024) | **0.130 (0.037, 0.223)** | 0.8 (-0.5, 2.1) |

Abbreviations: IDI, integrated discrimination improvement; NRI, net reclassification improvement.

The PRS reported here is the optimal PRS for any stroke (see **Table 1** for details). Numbers in the brackets represent the 95% confidence intervals, which were calculated by 100 bootstrap replications using the BCa method in Stata.

^a^ Participants with 10-year risk > 10% were grouped into a high-risk group.

^b^ When calculating relative IDI, cases were defined as participants who developed ischemic stroke or hemorrhagic stroke within 10 years of follow-up; non-cases were defined as those who were followed up for more than 10 years, including participants who developed ischemic stroke or hemorrhagic stroke after 10 years.

# References

[1] Lambert SA, Gil L, Jupp S, et al. The Polygenic Score Catalog as an open database for reproducibility and systematic evaluation. *Nat Genet.* 2021;53(4):420-425.

[2] Yang S, Li C, Hu Y, et al. gwasfilter: an R script to filter genome-wide association study. *Chin J Epidemiol.* 2021;42(10):1876-1881.

[3] Malik R, Chauhan G, Traylor M, et al. Multiancestry genome-wide association study of 520,000 subjects identifies 32 loci associated with stroke and stroke subtypes. *Nat Genet.* 2018;50(4):524-537.

[4] Sakaue S, Kanai M, Tanigawa Y, et al. A cross-population atlas of genetic associations for 220 human phenotypes. *Nat Genet.* 2021;53(10):1415-1424.

[5] Purcell S, Neale B, Todd-Brown K, et al. PLINK: a tool set for whole-genome association and population-based linkage analyses. *Am J Hum Genet.* 2007;81(3):559-575.

[6] Vilhjalmsson BJ, Yang J, Finucane HK, et al. Modeling Linkage Disequilibrium Increases Accuracy of Polygenic Risk Scores. *Am J Hum Genet.* 2015;97(4):576-592.
